# The Consent Process for Elective Hip and Knee Arthroplasty: Does Information on Handwritten Forms Meet Prescribed Standards?

**DOI:** 10.7759/cureus.23560

**Published:** 2022-03-28

**Authors:** Anirudh Sharma, Osasumwen Adelowo, Santosh Bindumadhavan, Naufal Ahmed, Amir-Reza Jenabzadeh

**Affiliations:** 1 Trauma & Orthopaedics, Hinchingbrooke Hospital, North West Anglia NHS Foundation Trust, Huntingdon, GBR

**Keywords:** british orthopaedic association, knee arthroplasty, hip arthroplasty, consent forms, informed consent

## Abstract

Introduction

The process of informed consent is vital, not only to good clinical practice and patient care, but also to avoid negligence and malpractice claims. Elective hip and knee arthroplasty numbers are increasing globally, and the British Orthopaedic Association (BOA) has endorsed standards for obtaining written consent for these procedures. Many centres in the United Kingdom and globally, use handwritten consent forms to document informed consent, leaving open the potential for missing out important procedure and risk-related information. Our study aimed to assess whether information on handwritten consent forms was compliant with BOA standards for elective arthroplasty of the hip and knee.

Methods

We retrospectively reviewed 70 handwritten consent forms, across theatre lists of 12 arthroplasty consultants at our elective arthroplasty centre. These included 35 forms each for hip and knee arthroplasty respectively. We compared the information on these forms to the standards prescribed by the BOA. We assessed compliance of the forms with common, less common and rare risks of hip and knee replacement, as described by the BOA. We also noted the designation of the person filling out the form (consultant, registrar or nurse practitioner) and whether this affected information on the form. We assessed the forms for legibility issues, and whether the setting (clinic/pre-operative ward) affected information on the form.

Results

None of the 70 forms reviewed achieved full compliance with BOA standards. When assessed for common risks of hip and knee arthroplasty, the number of compliant forms was 25.7% and 42.8%, respectively. None of the forms mentioned all rare risks of either hip or knee arthroplasty. We identified legibility issues in 12 of 70 (17.1%) forms. There was no significant difference in information written on forms filled out by consultants, registrars or nurse practitioners, or between forms filled out in the clinic versus those on the pre-operative ward.

Conclusion

Handwritten forms lack compliance with prescribed standards for written informed consent in elective hip and knee arthroplasty. Ideally, a pre-written consent form should be used, but with the option of adding information individually tailored to the patients’ background. This ensures that good clinical practice is optimally followed, and reduces the potential risk of any litigation.

## Introduction

The consent form has gained much importance as litigation associated with surgical procedures continues to increase, and can prove to be the most important document in many cases of malpractice litigation or negligence claim. While hip and knee arthroplasty is known to have excellent outcomes in terms of improvement in patients’ quality of life, there are known complications, which can result in varying degrees of morbidity. A study from 2013 on litigation following hip and knee arthroplasty in the National Health Service (NHS) in the United Kingdom (UK) shows that claims to a value of £62.5 million were made associated with hip and knee replacement [1]. Harrison et al. [2] have shown that 4% of claims of surgical negligence are related to consent and average about £40,000 per claim. Indeed, discussing risks and benefits with the patient, tailored individually to their background, rather than generic risks, is the most vital part of the consent process, and is included in the General Medical Council’s (GMC) good medical practice [3].

At our institution, as in many other NHS Trusts, the standard practice is for the written consent form to be signed by the patient on the morning of surgery [4]. Some surgeons prefer to have the consent form signed in clinic itself, after having listed the patient for surgery. The consent form used in many NHS Trusts is a generic form with spaces for the doctor taking consent, to write down the procedure, intended benefits and risks of the proposed surgery. This information is discussed in detail with the patient and the patient’s signature obtained on the form. In this setting, there are a number of factors that can cause the consent form to be inadequately filled out. Firstly, the documentation on the consent form may vary depending on the medical professional obtaining consent namely consultants, registrars, senior house officers (SHO) and nurse practitioners. Secondly, the time constraints of reviewing multiple patients on the pre-operative ward on the morning of surgery can lead to sub-optimal documentation. Indeed, a study has shown that consent obtained in a clinic setting is superior with regards to protection against a lawsuit, over one obtained in a pre-operative setting [5]. Thirdly, there can be issues with legibility with handwritten forms, which can result in information being misread or unable to be read. Fourthly, there can be variability in terminology, with different medical terms or abbreviations used on the form, which leaves open the possibility of this information being construed as insufficient or confusing in a court of law.

For the above-mentioned reasons, the British Orthopaedic Association (BOA) endorsed consent forms made available on www.orthoconsent.com as a standard guideline for obtaining written consent for orthopaedic procedures [6]. Forms for a range of orthopaedic procedures, both trauma and elective, are made available on the website and include comprehensive information on the procedure. The consent forms for hip and knee arthroplasty classify the risks based on their incidence as common (2-5%), less common (1-2%) and rare (<1%). We aimed to assess the quality and comprehensiveness of handwritten consent forms for elective hip and knee arthroplasty, in relation to prescribed BOA standards, to identify whether a change of practice is necessary. This knowledge would be useful for the orthopaedic community at large, and all institutions across the globe where handwritten forms are still the standard.

## Materials and methods

We retrospectively reviewed 70 consent forms - 35 each for primary hip and knee arthroplasty respectively, from an eight-week period at our institution, which is an elective orthopaedic centre and part of a District General Hospital (DGH) in the UK. These numbers ensured that we covered all consultants’ lists that run at our hospital over a two-week repeating rota, and that the data gathered would accurately reflect practice of the 12 arthroplasty consultants practicing at our institution. Revision arthroplasty procedures were excluded. We noted the designation of the medical professional taking consent (consultant, registrar, SHO or nurse practitioner). Two of the authors (AS and OA) assessed the forms for legibility issues. If both observers found it impossible to read one or more words on the form, it was marked as having concerns with legibility. We noted the number of forms filled out prior to the day of surgery in clinic by noting the date of filling out the form.

We assessed the consent forms to see how many of these mentioned all the risks as per the BOA standards. We further assessed the percentage of compliance of the forms with regards to common, less common and rare risks as shown in Table 1.

**Table 1:** The common, less common and rare risks for hip and knee arthroplasty, as mentioned on the BOA endorsed consent forms BOA: British Orthopaedic Association [6]

Frequency of Complication (Incidence)	Total Hip Arthroplasty	Total Knee Arthroplasty
Common (2-5%)	Blood clots (Deep Vein Thrombosis)	Pain
	Bleeding	Bleeding
	Pain	Blood Clots (Deep Vein Thrombosis)
	Prosthesis wear/loosening	Knee Stiffness
	Altered leg length	
	Dislocation	
Less Common (1-2%)	Infection	Infection
Rare (<1%)	Altered wound healing	Prosthesis Wear
	Nerve damage	Altered Leg Length
	Bone damage/Fracture	Altered Wound Healing
	Blood vessel damage	Joint Dislocation
	Pulmonary embolism	Nerve damage
	Death	Bone damage/Fracture
		Blood Vessel Damage

We used a student's t-test to note any statistical difference in the information on consent forms written up by consultants, registrars and nurse practitioners. The same was assessed for forms filled out in clinic versus those filled on the morning of surgery.

This study was registered with the hospital’s clinical audit committee and approval by the institutional ethics committee was not required for this review.

## Results

We reviewed a total of 70 consent forms, 35 each for hip and knee arthroplasty. Medical professionals filling out the consent form were found to include consultant orthopaedic surgeons, orthopaedic registrars and orthopaedic nurse practitioners. No consent forms were found to be filled out by an SHO. The distribution is shown in Table 2.

**Table 2:** Number of forms filled out by consultants, registrars and nurse practitioners

	Hip Arthroplasty	Knee Arthroplasty
Consultant	20	12
Registrar	14	20
Nurse Practitioner	1	3
Total	35	35

We identified legibility issues in 12 of 70 (17.1%) forms. Seventeen consent forms were written out in clinic prior to surgery (24.3%) and the rest were written on the morning of surgery on the ward.

We found that none of the consent forms for either hip or knee arthroplasty completely adhered to the BOA standards. The percentage compliance of the forms with regards to common, less common and rare risks is detailed in Table 3.

**Table 3:** Number and percentage of consent forms mentioning all risks described as common, less common and rare by the BOA endorsed consent form BOA: British Orthopaedic Association

	Hip Arthroplasty	Knee Arthroplasty
Common	9 (25.7%)	15 (42.8%)
Less Common	35 (100%)	35 (100%)
Rare	0 (0%)	0 (0%)

For hip arthroplasty, the ‘common’ risk most frequently omitted on the forms was ‘pain’ which was absent from 54.2% forms. This was followed by ‘bleeding’ absent from 28.6% forms, wear (17.1%) and leg length discrepancy (11.4%). Infection, classified as the only ‘less common’ risk was mentioned in 100% forms. Among the ‘rare’ risks, the most commonly omitted was ‘altered wound healing’ which was mentioned only on one form and omitted in 97.1% forms. This was followed by ‘death’ omitted in 42.8%, and ‘fracture’ omitted in 14.2% forms.

For knee arthroplasty, the ‘common’ risk most frequently omitted on the form similarly was ‘pain’ - absent from 37.1% forms. Bleeding and deep vein thrombosis/pulmonary embolism were mentioned in 97.1% forms and stiffness was mentioned in 94.3% forms. Similar to hip arthroplasty, ‘infection’ was mentioned in 100% forms. The most common rare complication omitted was altered leg length (97.1%), wound complications (94.3%) and dislocation (91.4%). Six forms failed to mention a periprosthetic fracture (17.1%).

We noted the number of omitted risks on the forms filled out by consultants, registrars and nurse practitioners respectively, and the mean number of omissions is shown in Table 4.

**Table 4:** Mean number of omitted risks by consultants, registrars and nurse practitioners

	Hip Arthroplasty	Knee Arthroplasty
Consultant	2.6	3.8
Registrar	2.8	3.8
Nurse Practitioner	2	3.7

There was no statistical difference on a t-test found between the number of omissions made by consultants and registrars (p - 0.38 for hip arthroplasty forms and p - 0.46 for knee arthroplasty forms). There was also no difference in the number of omissions on the forms filled out in clinic and those on the morning of surgery (p - 0.11).

## Discussion

Our results indicate that generic handwritten consent forms for elective hip and knee arthroplasty fall short of standard guidelines endorsed by the BOA. Hence, when used solely as evidence of informed consent, missing information on the forms can be held against the responsible surgeon in a court of law. It is imperative, therefore, to either use a standardized pre-printed consent form (with the option of adding further information bespoke to patient needs), or to document clearly on a clinic letter, the risks that were informed to the patient during the consultation.

Obtaining consent has been described as a process rather than a single event of getting a form signed by the patient [7,8]. The NHS Wales ‘Good Practice in Consent Implementation Guide’ [7] advises that for elective procedures, patients should have prior information of the content of their consent form, and must have a copy of the documentation of their consent. This emphasizes that all risks and benefits of the procedure are discussed with the patient in the clinic setting, rather than a single-step process of a signature on a form immediately prior to surgery. It has indeed been shown that consent obtained in a clinic setting reduces the risk of litigation, as compared to that obtained in the immediate pre-operative period [5]. Tonge et al. published an audit cycle, which showed that the quality of consent improved significantly with dedicated consenting clinics [9]. There is also evidence that a good consent process in clinic reduces pre-operative anxiety in patients [10].

Previous studies have also shown that handwritten consent forms in orthopaedics often have insufficient procedure-related information [11,12]. Our findings indicate that the designation and seniority of the person filling out the form did not improve information on the form, showing that it is not a lack of knowledge, but rather factors of time constraints and recall, which may be responsible. Studies have recommended using standardized pre-written consent forms to ensure comprehensive information is provided to patients [11,12]. These have also been shown to improve patients’ retention of information and understanding of the procedure [4].

However, it must be remembered that the addition of bespoke patient-specific information is vital to the consent process as highlighted in the Montgomery vs Lanarkshire case from 2015, which was seen as a landmark case changing the outlook towards the process of obtaining informed consent [13,14]. Therefore, while standardized forms help to serve as a good template, these should not be seen as a ‘one form fits all’ solution. Specific risks, for example, those posed by obesity, pathology such as hip dysplasia, implant longevity in a younger patient, etc. must always be specifically discussed and mentioned in the form or clinic letter.

Although our study is limited in its numbers, it reflects the practice of a wide number of individuals, and is representative of similar practices followed in many other hospitals worldwide where handwritten consent forms are used.

## Conclusions

We recommend that standardized pre-printed consent forms based on BOA guidelines are used for the consent process, and these should have additional spaces for the addition of individually tailored patient-specific risks. The form should be signed in clinic and the patient should have a period of time to re-read the form, consider all information, and ask any further questions on the day of surgery. This ensures a robust consent process and the patient receiving comprehensive and timely information on their procedure.

## References

[1] McWilliams AB, Douglas SL, Redmond AC, Grainger AJ, O'Connor PJ, Stewart TD, Stone MH (2013). Litigation after hip and knee replacement in the National Health Service. Bone Joint J.

[2] Harrison WD, Narayan B, Newton AW, Banks JV, Cheung G (2015). Litigation costs of wrong-site surgery and other non-technical errors in orthopaedic operating theatres. Ann R Coll Surg Engl.

[3] Jaques H (2013). GMC publishes new version of Good Medical Practice. BMJ.

[4] Barritt AW, Clark L, Teoh V, Cohen AM, Gibb PA (2010). Assessing the adequacy of procedure-specific consent forms in orthopaedic surgery against current methods of operative consent. Ann R Coll Surg Engl.

[5] Bhattacharyya T, Yeon H, Harris MB (2005). The medical-legal aspects of informed consent in orthopaedic surgery. J Bone Joint Surg Am.

[6] Atrey A, Leslie I, Carvell J, Gupte C, Shepperd JA, Powell J, Gibb PA (2008). Standardised consent forms on the website of the British Orthopaedic Association. J Bone Joint Surg Br.

[7] (2022). Good practice in consent implementation guide: consent to examination or treatment. DH.

[8] (2022). Consent: patients and doctors making decisions together. https://www.gmc-uk.org/-/media/documents/consent-patients-and-doctors-making-decisions-together-2008---2020_pdf-84769495.pdf?la=en.

[9] Tonge XN, Crouch-Smith H, Bhalaik V, Harrison WD (2021). Do we achieve the Montgomery standard for consent in orthopaedic surgery?. Br J Hosp Med (Lond).

[10] Torres-Lagares D, Heras-Meseguer M, Azcárate-Velázquez F, Hita-Iglesias P, Ruiz-de-León-Hernández G, Hernández-Pacheco E, Gutiérrez-Pérez JL (2014). The effects of informed consent format on preoperative anxiety in patients undergoing inferior third molar surgery. Med Oral Patol Oral Cir Bucal.

[11] Jeyaseelan L, Ward J, Papanna M, Sundararajan S (2010). Quality of consent form completion in orthopaedics: are we just going through the motions?. J Med Ethics.

[12] Heylen J, Antoniou V, Roberts J, Kemp O, Morris J, Vats A (2021). Assessing the quality of consent in elective hip and knee arthroplasty: do modern orthopaedic surgeons make the cut?. J Clin Orthop Trauma.

[13] Chan SW, Tulloch E, Cooper ES, Smith A, Wojcik W, Norman JE (2017). Montgomery and informed consent: where are we now?. BMJ.

[14] Dyer C (2015). Doctors should not cherry pick what information to give patients, court rules. BMJ.

